# Role of extracellular vesicles secretion in paclitaxel resistance of prostate cancer cells

**DOI:** 10.20517/cdr.2022.26

**Published:** 2022-06-21

**Authors:** Ashish Kumar, Pawan Kumar, Mitu Sharma, Susy Kim, Sangeeta Singh, Steven J. Kridel, Gagan Deep

**Affiliations:** ^1^Department of Cancer Biology; Atrium Health Wake Forest Baptist, Winston-Salem, NC 27157, USA.; ^2^Division of Pathology, Indian Veterinary Research Institute, Izatnagar, Bareilly, UP 243122, India.; ^3^Wake Forest Baptist Comprehensive Cancer Center, Atrium Health Wake Forest Baptist, Winston-Salem, NC 27157, USA.

**Keywords:** Prostate cancer, extracellular vesicles, GW4869, chemoresistance, paclitaxel

## Abstract

**Aim:** The development of chemotherapy resistance is the major obstacle in the treatment of advanced prostate cancer (PCa). Extracellular vesicles (EVs) secretion plays a significant role among different mechanisms contributing to chemoresistance. Hence, inhibition of EVs release may increase the efficacy of chemotherapeutic drugs against PCa.

**Methods:** Paclitaxel (PTX) resistant PCa cells (PC3-R and DU145-R) were treated with GW4869, a known exosome biogenesis inhibitor. EVs were isolated from the conditioned media by ExoQuick-based precipitation method and characterized for concentration and size distribution by nanoparticle tracking analysis. The effect of GW4869 treatment on the survival and growth of PCa cells was assessed by MTT, and colony formation assays *in vitro*, and ectopic PC3-R xenografts in male athymic nude mice *in vivo*. The effect of other EV biogenesis inhibitors, imipramine and dimethyl amiloride (DMA), treatment was also analyzed on the survival of PC3-R cells.

**Results:** GW4869 (10-20 µM) treatment of PTX resistant PCa cells significantly reduced the release of small EVs (50-100 nm size range) while increasing the release of larger EVs (> 150 nm in size), and inhibited their clonogenicity. Moreover, GW4869 (5-20 µM) treatment (24-72h) significantly inhibited the survival of PC3-R cells in a dose-dependent manner. We observed a similar growth inhibition with both imipramine (5-20 µg/mL) and DMA (5-20 µg/mL) treatment in PC3-R cells. Furthermore, GW4869 treatment (IP) in mice bearing PC3-R xenografts significantly reduced the tumor weight (65% reduction, *P* = 0.017) compared to the vehicle-treated control mice without causing any noticeable toxicity.

**Conclusion:** Inhibiting the release of EVs could sensitize the resistant PCa cells to chemotherapy.

## INTRODUCTION

Prostate cancer (PCa) is one of the most predominant types of cancer in men, accounts for 26% of all cancer cases, and remains one of the leading causes of death^[1]^. Most of the PCa cases show an indolent stage with no or less risk of mortality; however, many patients develop intermediate or high-risk locally advanced or metastatic phenotype. It is well established that the growth and progression of the PCa are dependent on androgen; thus, androgen deprivation therapy (ADT) is the most accepted practice as the first-line treatment for metastatic /disseminated PCa in the clinical setting^[2,3]^. Despite the initial response to the ADT, most patients develop castration-resistant PCa (CRPC), characterized by increased serum levels of prostate-specific antigen (PSA) and increased growth and metastasis of primary tumor, leading to disease aggressiveness despite castrate serum levels of testosterone^[4,5]^. Treatment of patients with CRPC is one of the major challenges for the development of effective treatment therapy for PCa. Many clinical trials have demonstrated the effectiveness of taxol (paclitaxel and its semisynthetic analog docetaxel) for the treatment of CRPC patients due to its effectiveness in prolonging the overall survival^[6-8]^. Docetaxel has already been approved by the United States FDA for the treatment of CRPC patients.

Paclitaxel (PTX) was the first member of the taxane family approved for cancer chemotherapy. PTX is used in the treatment of CRPC (in combination with ADT) and possesses anti-neoplastic properties by promoting and stabilizing the polymerization and assembly of tubulin required for microtubule formation during the cell cycle, which ultimately causes cell cycle arrest leading to apoptosis^[9]^. After multiple treatment schedules with PTX, cancer cells develop resistance that leads to a poor prognosis. The development of drug resistance by cancer cells is one of the major challenges in cancer therapy. However, the mechanism of drug resistance is largely unknown but often mediated by insufficient availability of the drug to the target cell. Many mechanisms which are adopted by cancer cells to develop drug resistance have been demonstrated, including drug efflux by membrane-bound efflux proteins, increased interstitial fluid pressure that impairs drug uptake, acidic extracellular microenvironment and hypoxia, hyperglycemia, cellular rewiring, altered drug metabolism, and mutation in drug’s target^[10-16]^. PTX resistant DU145 and PC3 cells have been shown to overexpress multiple drug resistance gene (MDR-1)-encoded P-glycoprotein and enhanced expression of F-actin polymerization^[17]^ as a mechanism to develop resistance against PTX. The direct expulsion of the anticancer drug through extracellular vesicles (EVs) may contribute crucially to the development of chemoresistance. In an earlier study, Shedden *et al*. demonstrated the efflux of anticancer drug doxorubicin through EVs, hence preventing the drug from reaching its target site and also decreasing the available drug concentration for its anti-neoplastic effect^[18]^. Moreover, it has also been reported that the EVs isolated from drug resistant-cancer cells can efficiently transfer proteins involved in drug efflux pumps to the drug-sensitive cells resulting in the acquisition of MDR^[19,20]^. Several studies have shown the involvement of EVs released from drug-resistant cells in transmitting resistance to the other cells (reviewed in^[21]^).

EVs are membranous vesicles released by all cell types and can contain nucleic acids, lipids, and proteins as cargos, which are representative of the secretory cell and its biological behavior^[22-24]^. EVs are classified into exosomes (~30-150 nm), ectosomes/microvesicles (100-1000 nm), apoptotic bodies (1-5 μM), and large oncosomes (1-10 μM) on the basis of their size and released pathway^[22,25]^. The role of EVs in cell-to-cell communication in modifying tumor microenvironment, preparation of pre-metastatic niche, and helping the formation of metastatic foci at distant sites in cancer is well established. Many studies have also suggested the role of EVs in mediating drug resistance in cancer^[23,26]^. Inhibition of the EVs biogenesis or release by cancer cells may be helpful in preventing the metastasis and the spread of drug resistance to the sensitive cells.

Inhibition of EV biogenesis using specific inhibitors can be used to evaluate the involvement of EVs in cancer cell proliferation and metastasis. GW4869, dimethyl amiloride (DMA), and imipramine are known chemical compounds commonly used as inhibitors of the EV biogenesis/secretion^[27]^. GW4869 acts as a non-competitive inhibitor of membrane neutral sphingomyelinase (nSMase) enzyme, which is responsible for the generation of lipid ceramide through the hydrolysis of the membrane lipid sphingomyelin and inhibits the release of small EVs/exosomes^[28,29]^. Imipramine is a tricyclic antidepressant that also exhibits an inhibitory activity on acid SMase and inhibits the generation of ceramide^[27]^. DMA inhibits H^+^/Na^+^ and Na^+^/Ca^2+^ exchangers and prevents the calcium gradient establishment which is required for the release of exosomes from the cells^[30,31]^. Earlier, we have reported that GW4869 and DMA treatment inhibits the survival and clonogenicity of PCa cells under both normoxic and hypoxic conditions, as well as the survival of enzalutamide-resistant PCa cells^[31,32]^. Earlier studies have also suggested that GW4869 reduces the chemoresistance in the cancer cells by inhibiting the EV release^[33,34]^. Richards *et al*. reported that treatment of gemcitabine to cancer-associated fibroblasts causes increased exosome secretion, which ultimately increases the rate of proliferation in chemosensitive pancreatic epithelial cancer cells and their survival, subsequent treatment with GW4869 significantly reduced their survival^[34]^. In the present study, we reported the inhibitory effects of GW4869 on the survival of PTX-resistant PCa cells, both *in vitro* and *in vivo*. We also found that the concentration and size distribution of EV released by PTX-resistant PCa cells was significantly affected by the GW4869 treatment.

## MATERIALS AND METHODS

### Cell lines and reagents

In this study, we used the previously developed PTX resistant human PCa PC3 and DU145 cells^[35,36]^. PTX resistant PC3 (PC3-R) and DU145 (DU145-R) cells were maintained in RPMI1640 medium supplemented with 10% heat-inactivated FBS, 100 units/mL final concentration of penicillin-streptomycin (Gibco Laboratories, Gaithersburg, MD), and 0.2 µM final concentration of PTX (in DMSO). Cells were incubated at 37 °C in CO_2_ incubator at a 5% CO_2_ concentration (Heracell VIOS 160i; ThermoFisher, Waltham, MA). GW4869 was purchased from Sigma-Aldrich (St. Louis, MO) and 5-(N,N-Dimethyl) amiloride hydrochloride (DMA) from ThermoFisher (Waltham, MA), while imipramine was purchased from Selleckchem (Pittsburgh, PA).

### EVs isolation

Total EVs were isolated from both PC3-R and DU145-R cells after treatment with 10 and 20 µM doses of GW4869. Cell number was counted, and condition media was collected after 24 and 48 h following treatment with GW4869. Next, EVs were isolated from conditioned media using ExoQuick^TM^ (System Biosciences, Palo Alto, CA) precipitation method as described earlier^[37]^. Briefly, the cell culture conditioned media was first centrifuged at 500 *g* for 5 min, 2,000 *g* for 10 min, 10,000 *g* for 30 min at 4 °C to remove large-sized vesicles. Finally, EVs were isolated using ExoQuick following the manufacturer’s recommendations. EV pellet was dissolved in filtered Dulbecco’s phosphate-buffered saline (DPBS).

### Nanoparticle tracking analyses

The concentration and size distribution of the EVs were analyzed using Nanosight NS300 (Malvern Instruments, UK) as described earlier^[38]^.

### MTT assay

PC3-R cells (~1,000) were seeded in 96-well plates and, after 24 h, treated with different doses of GW4869 (5, 10, and 20 µM), DMA (5, 10, and 20 µg/mL), and imipramine (5, 10, and 20 µg/mL). The MTT assay was performed 24, 48, and 72 h after treatment. Briefly, 20 µL MTT (5 mg/mL in PBS) was added to each well and incubated in the dark for 2 h, and then 200 µL of DMSO was added to dissolve the formazan crystals. Finally, absorbance was measured at 560 and 650 nm.

### Clonogenic assay

PC3-R and DU145-R cells (200 cells per well) were seeded in 6-well plates and treated with 10 and 20 µM doses of GW4869. After 6-7 days of treatment, colonies (≥ 50 cells) were counted under a microscope. Thereafter, cells were fixed in methanol, stained with crystal violet solution, dried, and photographs were captured. 

### Tumor Xenograft

All mice were housed and all experiments performed in accordance with the protocol approved by the Institutional Animal Care and Use Committee (IACUC) at Wake Forest University Health Sciences (Winston-Salem, NC). Male athymic nude mice (*nu/nu*) were purchased from Envigo (Indianapolis, IN) at 4-6 weeks of age and were given ad libitum food and water on a 12-h light-dark cycle.

PC3-R cells were collected on the day of injection and resuspended in serum-free media. An equal volume of Matrigel matrix (Corning, Bedford, MA) was added to the cells and kept on ice. Mice were anesthetized with Isoflurane at 2%-4%. Using a 1cc syringe with a 27g needle (BD, Franklin Lakes, NJ), ~2.1 × 10^6^ cells were injected subcutaneously on each flank of all the mice. As soon as the tumors were visible, they were measured twice a week with calipers, and volume was calculated as Width (mm)^2^ × Length (mm) × 0.52 = Volume (mm)^3^. At the time when the tumors were approximately 50-100 mm^3^, mice were divided into two groups. Mice were treated with either GW4869 (Sigma, St. Louis, MO) or vehicle [Vehicle control (VC)], 5% DMSO in 0.9% Sodium Chloride (Baxter, Deerfield, IL). Mice were injected intraperitoneal (IP) with 2.5 mg/kg GW4869 or vehicle (200uL) 6 days a week for 21 days, and then the dose of GW4869 was increased to 5.0 mg/kg. Throughout the experiment, the body weight of each animal was regularly measured. Any mouse with xenograft volume approaching the size limit or other parameters (e.g., necrosis) defined by IACUC was sacrificed, and tissues were collected. At the end of the experiment, animals were sacrificed using CO2 asphyxiation, and blood and tumors were harvested. Plasma was isolated from the blood and stored at -80 °C until further use.

### Tissue processing and immunohistochemistry

Tumor tissues were processed, and 5 µm formalin-fixed and paraffin-embedded (FFPE) sections were analyzed for CD63 expression by processing and immunohistochemistry (IHC) as described by us previously^[39]^. CD63 primary antibody was purchased from ThermoFisher (Waltham, MA) and detection system (ImmPRESS-AP horse anti-rabbit IgG polymer detection kit) with secondary antibody from Vector laboratories (Burlingame, CA). All the immunostained slides were scanned by NanoZoomer (Hamamatsu, Japan) at 20×. The CD63 immunostaining scoring was conducted manually by evaluating the intensity of staining and percentage of stained cells: intensity was given scores of 0-3 (0 = no staining, 1 = weak staining, 2 = moderate staining, 3 = intense staining), and the percentage of immunopositive cells was given scores 0%-100%. The 2 scores were multiplied to obtain the final IHC score (between 0-300). 

### EVs isolation from plasma

Isolation of total EVs from mice plasma was performed as described by us previously^[38,40]^. Briefly, plasma was diluted in PBS and centrifuged at 500 *g* for 5 min, 2,000 *g* for 10 min, followed by 10,000 *g* for 30 min at 4 ℃ to remove the larger sized vesicles. Finally, EVs were isolated using ExoQuick following the manufacturer’s recommendations (System Biosciences, Palo Alto, CA). EV pellet was dissolved in filtered DPBS.

### Western blotting

Briefly, 35 µL of EVs isolated from mice plasma were denatured directly in 5× loading buffer and separated using sodium dodecyl sulfate-polyacrylamide gel electrophoresis on a 12% Tris-glycine gel. The separated proteins were transferred onto nitrocellulose membrane, and after blocking with 5% non-fat milk powder (w/v) in Tris-buffered saline with tween (TBS-T, 10 mM Tris-HCl, pH 7.5, 100 mM NaCl, and 0.1% Tween 20), the fresh membrane or stripped membrane was probed with anti-syntenin (Abcam, Waltham, MA) or anti-CD63 (Invitrogen, Waltham, MA) or anti-GOLGA2 (Novus Biologicals, Littleton, CO) primary antibodies for overnight incubation at 4 ℃. Further, in each case, the membrane was washed 3 times with TBS-T and incubated with appropriate secondary antibodies before visualization by the ECL detection system. The autoradiograms/ bands were scanned and quantified using Image J (version 1.53e) using corresponding bands in Ponceau stained membrane as the loading control.

### Statistical analysis

Student’s *t* test (unpaired) was used to examine the statistical significance (*P* < 0.05) of differences between control and treatment groups.

## RESULTS

### Effect of GW4869 treatment on the EVs secretion by PC3-R cells

**Figure 1:**
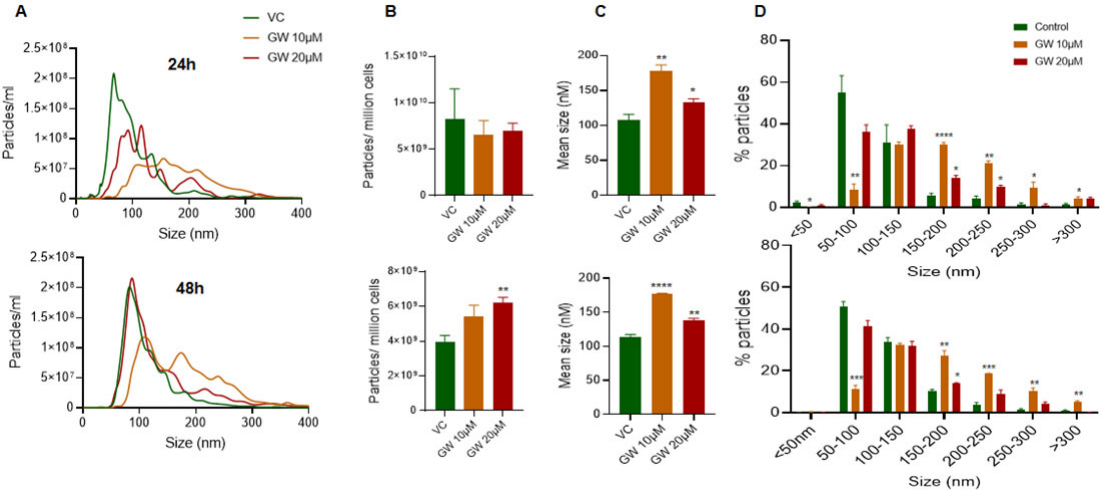
Characterization of EV concentration and size distribution following GW4869 treatment in PC3-R cells. EVs were isolated from the conditioned media of paclitaxel-resistant PC3-R cells following 24 h (upper panel) and 48 h (lower panel) of treatment with GW4869 (10-20 µM) and characterized for concentration and size distribution by NTA. (A) Concentration and size distribution of EVs isolated from DMSO (VC) or GW4869 (10 and 20 µM) treated cells are represented with green, orange, and red colors, respectively (*n* = 3). Each line represents the mean of three samples, and an average data of 5 videos of 30 sec each was used for each sample. (B-C) Total EV concentration per million cells and mean size are plotted. (D) Size distribution of EVs is presented as percent particles in the mentioned size range. Each bar represents mean ± SEM (*n* = 3). VC: Vehicle control; *P < 0.05, **P < 0.005, ***P < 0.0005, ****P < 0.0001.

PC3-R cells were treated with 2 different doses (10 µM and 20 µM) of GW4869, and conditioned media was collected after 24 and 48 h. A significant shift in the size distribution of EVs was observed at both 24 and 48 h, with a more noticeable difference at 10 µM dose [Figure 1A]. Though no difference in total particle number secreted per million cells was observed at 24 h, a significant increase in the particle number per million cells was observed at the higher dose of GW4869 with 48h of treatment [Figure 1B, lower panel]. We also noticed a significant increase in the mean size of EVs isolation at 24 and 48 h with both the doses, though the increase was more prominent with the 10 µM dose [Figure 1C]. Analysis of change in EVs in different size ranges showed that the proportion of EVs in size range 50-100 nm was significantly decreased, while the proportion of > 150 nm sized EVs increased significantly with 10 µM dose of GW4869 at both 24 h and 48 h [Figure 1D]. Treatment of PC3-R cells with a 20 µM dose of GW4869 resulted in a 35% reduction (though statistically non-significant) in the proportion of 50-100 nm sized EVs and increased the proportion of 150-200 nm and 200-250 nm sized EVs at 24 h time [Figure 1D, upper panel]. Furthermore, GW4869 at 20 µM dose marginally (statistically not significant) reduced the proportion of 50-100 nm sized EVs and increased 150-200 nm sized EVs at 48 h [Figure 1D, lower panel].

### GW4869 reduces the survival and clonogenicity of PC3-R cells

**Figure 2:**
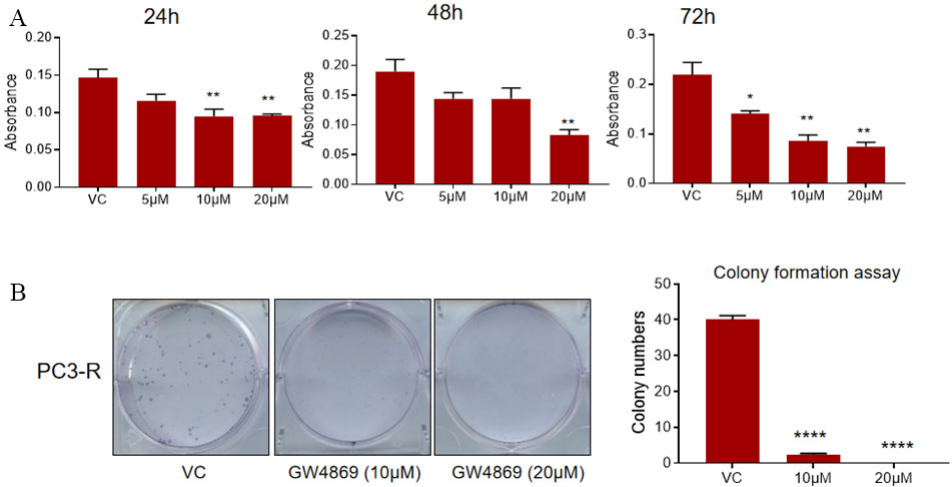
Effect of GW4869 treatment on the survival and clonogenicity of PC-R cells. (A) Paclitaxel-resistant PC3-R cells were treated with DMSO (VC) or GW4869 (5-20 µM), and cell viability was measured in MTT assay after 24, 48, and 72 h. Data are presented as mean ± SEM (*n *= 5 replicates per group). *P < 0.05, **P < 0.005. (B) Colony formation was measured in PC3-R cells after GW4869 treatment (10 and 20 μM) as described in the methods. Representative images are shown (left panel), and the colony number is presented as mean ± SEM (*n* = 3 replicates per group). ****P < 0.0001. VC: Vehicle control.

Next, an MTT assay was performed to assess the viability of the PC3-R cells after GW4869 (5-20 µM) treatment for 24-72 h. A significant reduction in cell viability was observed compared to control in treatment with a 20 µM dose at all the time points. We observed a decrease in cell viability with a 10 µM dose at either early (24 h) or late time (72 h); however, with a 5 µM dose, we only observed a statistically significant decrease at 72 h [Figure 2A].

Since PC3-R cells showed a prominent effect of GW4869 treatment mostly at later time points, a colony formation assay was performed to assess the effect on the clonogenicity of PC3-R cells following GW4869 treatment. In colony formation assay, a significant reduction in the number of colonies was observed in PC3-R cells at both 10 and 20 µM doses [Figure 2B].

### GW4869 administration inhibits the PC3-R xenograft tumor growth in nude mice

**Figure 3:**
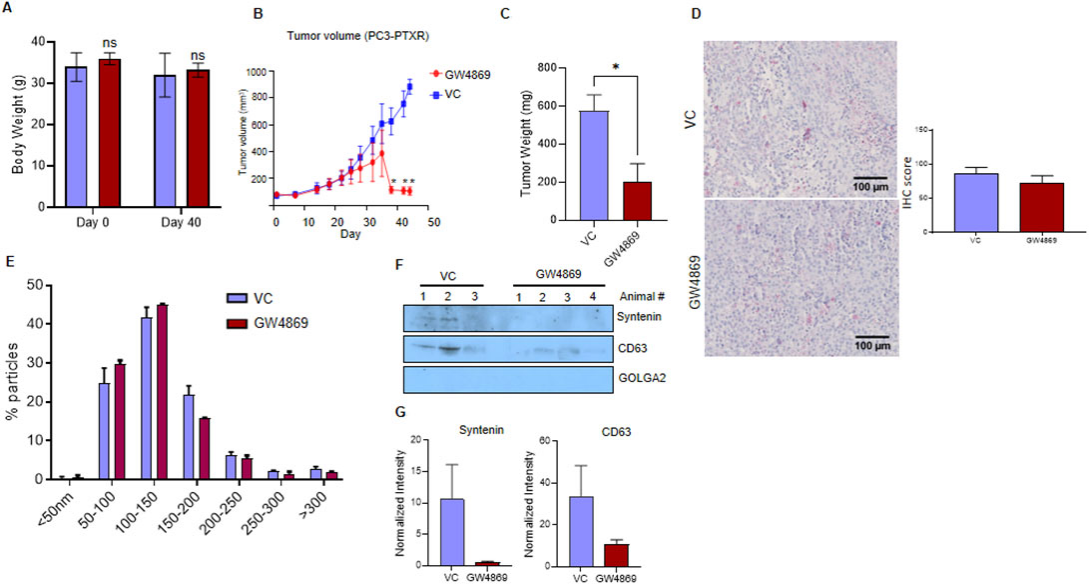
*In vivo* effect of GW4869 treatment on the growth of paclitaxel-resistant PC3-R cells’ xenografts. Male athymic nude mice were treated with vehicle or GW4869, and various study parameters were assessed as described in the methods. (A) Average body weight (mean ± SEM) at the start and day 40 of the study for control mice (*n* = 3) and GW4869 treated mice (*n* = 4) mice. (B) Average xenograft volume (mean ± SEM) in control and GW4869 treated mice survived at measured time points is presented. (C) In the end, mice were sacrificed, xenografts were excised, and their weight was measured. Average tumor weight in the control group (*n* = 6 xenografts) and the GW4869 group (*n* = 8 xenografts) is presented as mean ± SEM. Unpaired t test was used to calculate the statistical significance, *P = 0.017. (D) CD63 expression in xenograft tissues from the vehicle control (VC) group and GW4869 group (*n* = 6 each) was measured by IHC. For each image, ten random areas were analyzed for IHC scoring as described in the methods. Mean IHC scores are presented as mean ± SEM in the bar diagram. Representative images are shown. (E) EVs isolated from the plasma of control (*n* = 3) and GW4869 treated mice (*n* = 3) were analyzed by NTA, and the size distribution (mean ± SEM) of EVs was plotted as a percentage of total EVs. (F) EVs from VC (*n* = 3) and GW4869 treated (*n* = 4) mice were used for the analysis of syntenin, CD63, and GOLGA2 by Western blotting. Representative immunoblots are shown. (G) Densitometry analysis of syntenin and CD63 expression was performed and normalized with corresponding band intensity in Ponceau-stained membrane. The relative band intensity is presented as mean ± SEM.

The anti-tumorigenic potential of the GW4869 was evaluated in the nude mice by implanting PC3-R cells subcutaneously in two groups (control and GW4869). No significant change in the body weight of control and GW4869 administered mice was observed after 40 days [Figure 3A]. The tumor volume from the mice that survived at each time point was measured, and the animals that survived by the end of the study were sacrificed on the 44th day after PC3-R cells implantation. A statistically significant difference in tumor volume was observed compared to the control group towards the end of the experiment [Figure 3B]. The tumor tissue weight was also reduced significantly (65% reduction, *P *= 0.017) with GW4869 treatment compared to the control group [Figure 3C].

The expression of CD63 (typical small EVs/exosome marker) in tumor tissues was analyzed by IHC to identify the effect of GW4869 on EVs biogenesis in the tumor tissue. However, no significant difference in the CD63 expression was observed between control and GW4869 treated mice [Figure 3D]. Further, total EVs were isolated from the plasma of control and GW4869 treated animals and analyzed by NTA. No significant difference in the concentration and size distribution was observed between the control and GW4869 groups [Figure 3E]. However, Western blot analysis showed a 95% decrease in the expression of syntenin and about a 70% decrease in the expression of CD63 [Figure 3F and G]. GOLGA2 (Golgin A2) was used as a potential negative marker for EV cargo.

### DMA & imipramine treatment reduce the survival of PC3-R cell 

**Figure 4:**
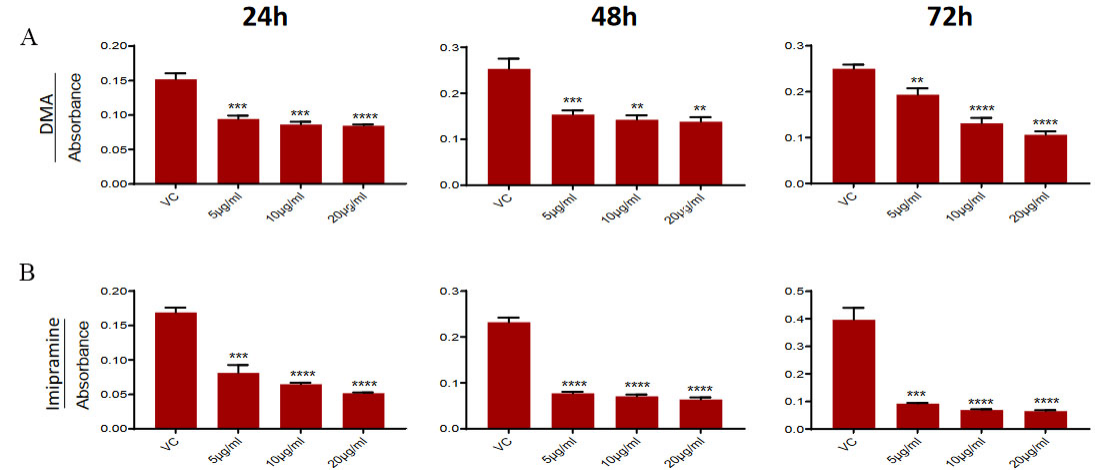
Effect of DMA and imipramine treatment on the survival of paclitaxel-resistant PC3-R cells. PC3-R cells were treated with (A) DMA (5-20 µg/mL) or (B) imipramine (5-20 µg/mL) for 24-72 h and analyzed for cell viability in the MTT assay. Data are presented as mean ± SEM (*n *= 5 replicates per group). **P < 0.005, ***P < 0.0005; ****P < 0.0001.

To confirm that the observed effect of GW4869 on PC3-R cells and tumor growth is mediated by inhibition of EVs secretion, we used two other compounds (DMA and imipramine), known to inhibit EV biogenesis [reviewed in^[27]^]. PC3-R cells were treated with different concentrations of DMA and Imipramine to evaluate their effect on cell viability by MTT assay. Treatment with different concentrations (5-20 μg/mL) of DMA showed a significant reduction in the viability of PC3-R cells at all time intervals [Figure 4A]. Similarly, imipramine treatment (5-20 μg/mL) also reduced the PC3-R cell’s survival at all-time intervals with all concentrations [Figure 4B].

### GW4869 treatment inhibits EVs secretion and clonogenicity of DU145-R cells

**Figure 5:**
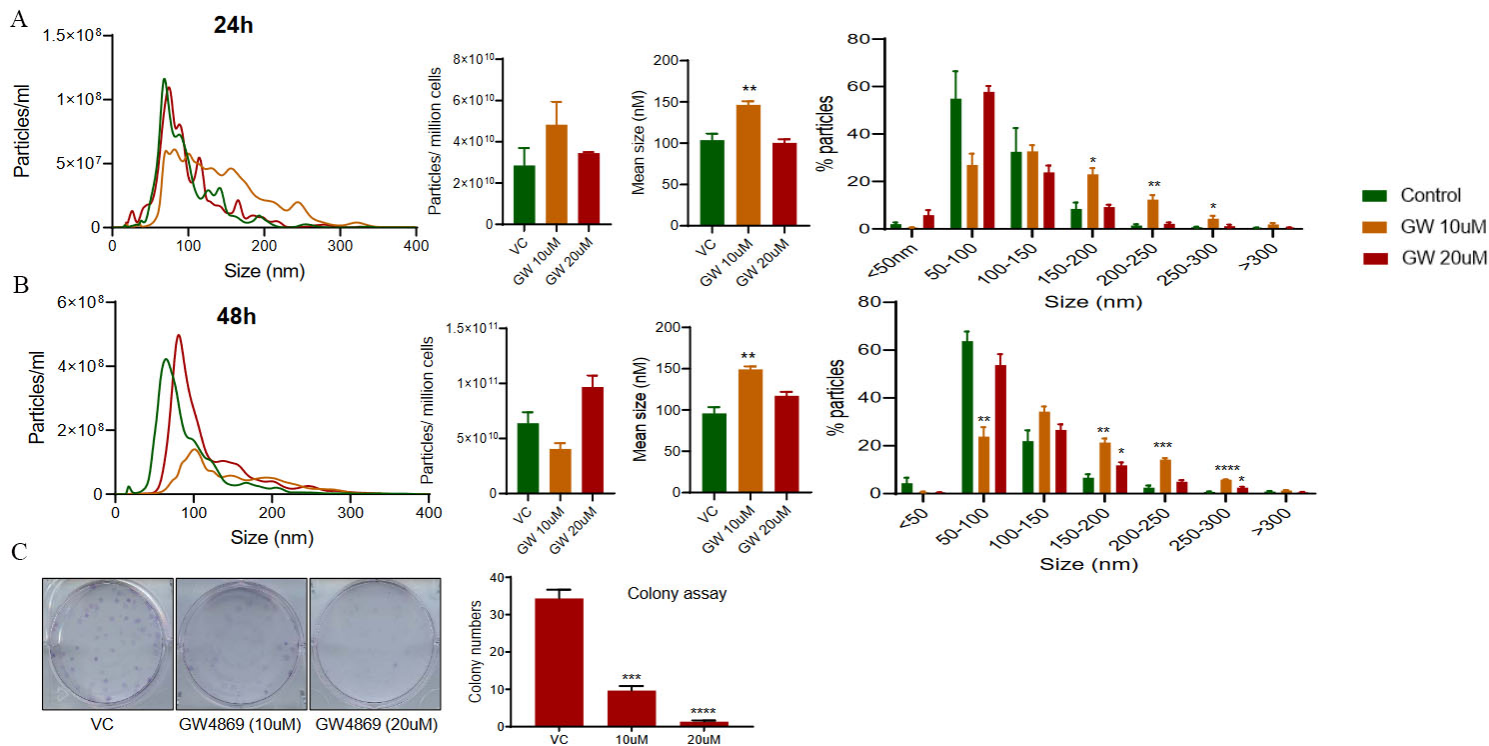
Effect of GW4869 treatment on paclitaxel-resistant DU145-R cells. EVs were isolated from the conditioned media of paclitaxel-resistant DU145-R cells following 24 h (upper panel) and 48 h (lower panel) of treatment with GW4869 (10-20 µM) and characterized for concentration and size distribution by NTA. (A-B) Each colored line in the left panel represents the mean of three samples, and an average data of 5 videos of 30 s each was used for each sample. Concentration (particles/mL)-size distribution, average EV concentration per million cells, average size, and percent particles for various size ranges are presented. Each bar represents mean ± SEM (*n* = 3). *P < 0.05, **P < 0.005 ***P < 0.0005 ****P < 0.0001. (C) Colony formation was measured in DU145-R cells after GW4869 treatment (10 and 20 μM) as described in the methods. Representative images are shown (left panel), and the colony number is presented as mean ± SEM (*n *= 3 replicates per group). ***P < 0.0005, ****P < 0.0001.

The effect of GW4869 treatment was also assessed on EVs secretion by PTX resistant DU145-R cells. GW4869 treatment did not significantly affect the particles per million cells at both 24 h and 48 h [Figure 5A and B]. We observed a significant increase in the mean size of the particles with a 10 μM dose at both 24 and 48 h, while no change was observed with a 20 μM dose [Figure 5A and B]. Consistent with PC3-R cells, GW4869 treatment reduced the proportion of 50-100 nm sized EVs by more than 50% (though statistically not significant) at 10 μM dose and increased the proportion of > 150 nm sized EVs at 24 h [Figure 5A]. The treatment with a 20 µM dose of GW4869 did not show any significant effect on EVs size at 24 h [Figure 5A]. We also observed a significant reduction in the proportion of 50-100 nm sized EVs with 10 µM dose of GW4869 and a statistically significant increase in 150-200 nm, 200-250 nm, and 250-300 nm sized EVs at 48 h. Further, a higher dose of GW4869 (20 µM) increased the proportion of 150-200 nm and 250-300 nm sized EVs after 48 h of treatment without significantly affecting the proportion of 50-100 nm sized EVs [Figure 5B].

The colony formation assay showed that GW4869 treatment (10 and 20 μM) resulted in a significant reduction in the number of colonies formed by DU145-R cells [Figure 5C]. 

## DISCUSSION

Chemotherapy is the major treatment option for CRPC; however, the acquisition of chemoresistance by PCa cells is considered as the main obstacle in the development of an effective anticancer strategy. Many molecular mechanisms that contribute to the development of chemoresistance in cancer cells have been suggested, including transporter pumps, altered metabolism, alteration in gene expression, epithelial to mesenchymal transition, cancer stemness, hypoxia, and acidic tumor microenvironment^[41-43]^. Drug-resistant cancer cells utilize EVs to transfer the transporter pumps and other biomolecules involved in pro-survival and anti-apoptotic pathways to the sensitive cells for propagation of chemoresistance^[23,44]^. Additionally, cancer cells can use EVs for direct loading of drugs and their efflux, which can undoubtedly contribute crucially to the development of chemoresistance in cancer cells. Using different cell line models, Shedden et al. have shown the physical incorporation of doxorubicin in EVs and its expulsion into the media^[18]^. It indicated that cancer cells secrete the EVs as an intrinsic mechanism to enable the resistant cells to survive under stressful or toxic environments. Further, it is well accepted that in physiological and biochemical stress conditions like hypoxia or an acidic environment, cancer cells secret higher amounts of EVs^[31,45,46]^ as a protective mechanism to remove metabolic and toxic waste. Treatment of PTX resistant PC3-R and DU145-R cells with GW4869 (a selective neutral sphingomyelin phosphodiesterase inhibitor) showed a specific reduction on 50-100 nm sized EVs, which are in the size range of exosomes/ small EVs, though increased the proportion of larger size (> 150 nm) EVs. The observation was in line with the previous report suggesting the treatment of human breast cancer cell line SKBR-3 with 5 µM GW4869 or siRNA against sphingomyelin phosphodiesterase 2/3 (also known as nSMase) resulted in a significant reduction of < 100 nm sized vesicles and increased quantities of vesicles with a size range of 100-200 nm; while overexpression of sphingomyelin phosphodiesterase-3 (also known as nSMase2) decreased the amount of larger-sized (100-200 nm) vesicles^[47]^. Interestingly, the increase in the EV fraction, following GW4869 treatment for 16 h, collected after 14,000 × *g* centrifugation (larger sized vesicles) was suggested to be stemming from plasma membrane representing MVs. Moreover, higher content of sphingomyelin in the MVs membrane was reported with respect to the overall cell membrane lipid composition, while no difference in EVs collected at 100,000 × *g* fraction (small EVs) was observed. Therefore, inhibiting the nSMase with GW4869 can interfere with the lipid composition, which may inhibit small EVs/exosome secretion but increase the secretion of larger EVs/MVs^[47]^. Interestingly, in line with our observation, the treatment of SKBR3 cells with GW4869 was shown to affect the loading of proteins (including syntenin) in EVs and carry less protein per EV^[47]^.

Several studies have indicated that EVs or exosome secretion can be inhibited by the use of GW4869, imipramine, and DMA^[28,31,32,48-52]^. Interestingly, treatment of PTX-resistant PCa cells with these known exosome biogenesis inhibitors resulted in a significant decrease in cell viability, especially in a long-term clonogenic assay. Similar inhibition of EVs secretion with GW4869 treatment leading to decreased growth of B16BL6 cells was reported earlier^[53]^. We have also previously reported that the treatment with GW4869 and DMA decreased the cell survival of the enzalutamide-resistant PCa cells^[32]^ as well as clonogenicity of PCa cells under both normoxic and hypoxic conditions^[31]^.

The study by Corcoran *et al*. has shown that docetaxel resistant variants of DU145 and 22Rv1 cells transfer the docetaxel resistance to sensitive cells (DU145, 22Rv1 and LNCaP) partly through MDR-1/P-gp transfer, suggesting the role of exosomes in the propagation of chemoresistance^[54]^. Moreover, other studies have reported the inhibition of EVs secretion in cell culture and/or suppression of tumor growth in mice after GW4869 treatment^[53,55-57]^. *In vivo* experiment in the present study indicated that GW4869 treatment decreases the tumor growth significantly without affecting the overall bodyweight of the mice. Although, we did not observe a significant change in the total EV number isolated from the plasma of these mice and CD63 expression in the xenograft tissues. However, we observed a strong decrease in the loading of known exosomal cargo proteins (syntenin and CD63). Earlier, Dinkins et al. have shown the reduction in protein concentration and reduced levels of Alix (a marker for exosomes/EVs) in EVs isolated from the mice serum treated with intraperitoneal injections of 100 µg GW4869 (~4 µg/g) daily for 5 days^[51]^. The effect of GW4869 treatment on the concentration of EVs was not measured in this study^[51]^. Similarly, intraperitoneal treatment of C57BL/6J mice with GW4869 (1.25 mg/kg) decreased airway-secreted EVs *in vivo*, as measured directly with NTA and indirectly with miRNAs expression^[58]^. Essandoh *et al*. also showed that treatment of wild-type mice with GW4869 at a dose of 2.5 µg/g of body weight reduced the levels of serum exosomes by 33%. Though, the concentration of exosomes was measured indirectly by acetylcholinesterase (AChE) activity assay^[59]^. Interestingly, in an established multiple myeloma (MM) mouse model, intraperitoneal injection of 1.25 µg/g GW4869 for 2 weeks suppressed MM tumor growth, though no association with nSMase expression and sensitivity to GW4869 was observed. Moreover, the molar levels of sphingomyelin decreased while ceramide increased in GW4869 treated MM cells for a specific duration, suggesting that the cytotoxic effect of GW4869 may not be through direct nSMase2 inhibition^[60]^. Overall, it appears that the dose and treatment duration of GW4869, as well as the cancer-type or study model, could also impact its effect on EV biogenesis and secretion in biofluids.

The mechanism by which GW4869 inhibits the survival and growth of the PTX-resistant PCa cells reported in this study could be partly through the inhibition of smaller EVs secretion (50-100 nm). The GW4869 mediated inhibition of the growth of the PTX resistant cells *in vitro* and *in vivo* indicated its usefulness in combating the chemoresistance in the PCa. The potential of the GW4869 as a combinational therapy to the PCa along with chemotherapeutic drugs needs to be explored further for the better management of chemoresistant PCa**.**

## DECLARATIONS

### Availability of data and materials

Raw data that support the findings of this study are available from the corresponding author upon reasonable request.

### Copyright

© The Author(s) 2022.
